# Dose‐Dependent Carbon‐Dot‐Induced ROS Promote Uveal Melanoma Cell Tumorigenicity via Activation of mTOR Signaling and Glutamine Metabolism

**DOI:** 10.1002/advs.202002404

**Published:** 2021-02-25

**Authors:** Yi Ding, Jie Yu, Xingyu Chen, Shaoyun Wang, Zhaoxu Tu, Guangxia Shen, Huixue Wang, Renbing Jia, Shengfang Ge, Jing Ruan, Kam W. Leong, Xianqun Fan

**Affiliations:** ^1^ Department of Ophthalmology Shanghai Ninth People's Hospital Shanghai JiaoTong University School of Medicine Shanghai 200011 China; ^2^ Shanghai Key Laboratory of Orbital Diseases and Ocular Oncology Shanghai 200011 China; ^3^ Department of Biomedical Engineering Columbia University New York NY 10027 USA; ^4^ State Key Laboratory of Oncogenes and Related Genes Institute for Personalized Medicine School of Biomedical Engineering Shanghai Jiao Tong University Shanghai 200030 China

**Keywords:** glutamine, metabolomics, mTOR, ROS, uveal melanoma

## Abstract

Uveal melanoma (UM) is the most common intraocular malignant tumor in adults and has a low survival rate following metastasis; it is derived from melanocytes susceptible to reactive oxygen species (ROS). Carbon dot (Cdot) nanoparticles are a promising tool in cancer detection and therapy due to their unique photophysical properties, low cytotoxicity, and efficient ROS productivity. However, the effects of Cdots on tumor metabolism and growth are not well characterized. Here, the effects of Cdots on UM cell metabolomics, growth, invasiveness, and tumorigenicity are investigated in vitro and in vivo zebrafish and nude mouse xenograft model. Cdots dose‐dependently increase ROS levels in UM cells. At Cdots concentrations below 100 µg mL^−1^, Cdot‐induced ROS promote UM cell growth, invasiveness, and tumorigenicity; at 200 µg mL^−1^, UM cells undergo apoptosis. The addition of antioxidants reverses the protumorigenic effects of Cdots. Cdots at 25–100 µg mL^−1^ activate Akt/mammalian target of rapamycin (mTOR) signaling and enhance glutamine metabolism, generating a cascade that promotes UM cell growth. These results demonstrate that moderate, subapoptotic doses of Cdots can promote UM cell tumorigenicity. This study lays the foundation for the rational application of ROS‐producing nanoparticles in tumor imaging and therapy.

## Graphical abstract

Uveal melanoma (UM) originates from melanocytes that are susceptible to malignant transformation by reactive oxygen species (ROS). Carbon dots (Cdots) with efficient ROS productivity dose‐dependently promote UM cell growth, invasiveness, and tumorigenicity at concentrations below 100 µg mL^−1^. Cdot‐induced ROS act as stimulatory signaling molecules to activate Akt/mammalian target of rapamycin signaling, accelerate glutamine metabolism, thus promoting UM cell growth.

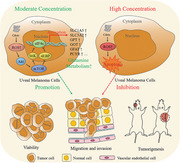

## 1 Introduction

Carbon dots (Cdots) have generated great interest as a new class of nanomaterial for use in medical imaging and targeted therapy applications due to their unique photophysical properties, facile surface functionalization, and low toxicity as well as their green synthesis due to high water solubility.^[^ ^1^ ^]^ Cdots have shown low systemic toxicity even at high doses in mice,^[^ ^2^ ^]^ but their effects on normal and tumor cell metabolism and growth have not been thoroughly characterized. Carbon‐based nanomaterials, including Cdots, nanotubes, graphene, and graphene oxide, have shown dose‐dependent cytotoxicity that can cause DNA and lysosomal damage and mitochondrial dysfunction, leading to apoptosis or necrosis.^[^ ^3^ ^]^ This cytotoxicity is attributed mainly to the production of reactive oxygen species (ROS),^[^ ^4^ ^]^ the byproducts of oxygen metabolism, which include superoxide anions, hydrogen peroxide, hydroxyl radicals, and hydroxyl ions. Exposure to carbon nanotubes causes various cell types, including human retinal pigment epithelial cells, fibroblasts, bronchial epithelial cells, and macrophages, to produce cytotoxic levels of ROS. Graphene and graphene oxide penetrate cell mitochondrial membranes and induce ROS production, leading to activation of mitogen‐activated protein kinase (MAPK) and transforming growth factor‐*β* (TGF‐*β*) signaling, disruption of mitochondrial function, and apoptosis.^[^ ^5^ ^]^ In macrophages, graphene quantum dots stimulate ROS production, leading to apoptosis or autophagy.^[^ ^6^ ^]^ In human fibroblasts, Cdots stimulate ROS production, causing reduced cell viability.^[^ ^7^ ^]^

**Scheme 1:**
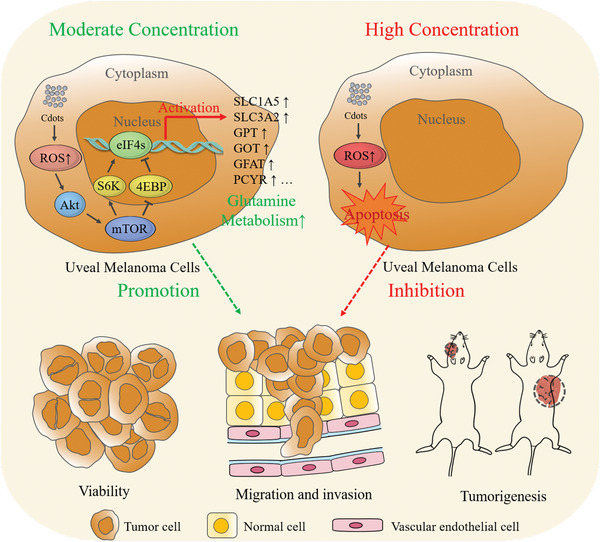
Schematic of the opposing Cdot‐concentration‐dependent effects on tumor cell progression and metastasis. At moderate Cdot concentrations, Cdot‐induced ROS promote tumor cell growth and invasiveness. At high Cdot concentrations, overwhelming ROS production causes tumor cell apoptosis.

ROS were originally considered a cytotoxic side product of tumor development that could be harnessed to kill tumor cells.^[^ ^8^ ^]^ However, some studies have shown the opposite—that ROS can promote tumor formation, malignant transformation, and chemotherapy resistance.^[^ ^9^ ^]^ Oxidative stress occurs when the balance between ROS and antioxidants such as ascorbic acid and glutathione is disrupted and is found in many types of cancer, including melanoma, hepatocellular carcinoma, glioma, and cancers of the breast, pancreas, bladder, colon, lung, and prostate.^[^ ^10^ ^]^ Elevated ROS can cause DNA damage, genetic mutation, activation of the c‐Ha‐ras‐1 proto‐oncogene, and inactivation of the p53 tumor suppressor gene.^[^ ^11^ ^]^ ROS stimulate tumor cell growth by activating PI3K/Akt and MAPK signaling and alter the expression of tumor‐related transcription factors, including AP‐1, nuclear factor κB(NF‐*κ*B), Nrf2, hypoxia inducible factor‐1*α* (HIF‐1*α* ), and p53.^[^ ^12^ ^]^ ROS stimulate tumor cell migration signaling in bladder cancer^[^ ^13^ ^]^ and are associated with invasion and metastasis in lung cancer.^[^ ^14^ ^]^ ROS generated by endogenous nicotinamide adenine dinucleotide phosphate (NADPH) oxidase, lysyl oxidase, and the mitochondrial electron transport chain activate tumor cell integrins that are key to the invasion of peripheral tissues.^[^ ^15^ ^]^ It is therefore important to study the effects of Cdot‐induced ROS on tumor cell metabolism, growth, and tumorigenicity (**Scheme** **1**).

Uveal melanoma (UM) is the most common intraocular malignant tumor in adults. Almost 50% of UM patients eventually have distant metastases and the 5‐year survival rate for metastatic UM is as low as 35%. The median overall survival for patients with metastases is 6–12 months. UM originates in melanocytes in the choroid, ciliary body, or iris. Melanocytes generate ROS in response to exposure to ultraviolet light, increasing the risk of melanoma.^[^ ^16^ ^]^ Population‐based studies have established a relationship between ROS levels and UM incidence. Two clinical studies showed that UM incidence is highest in light‐colored eyes,^[^ ^17^ ^]^ and a meta‐analysis (ten studies, 1732 cases) found that a gray or blue iris is a risk factor for developing UM.^[^ ^18^ ^]^ Melanocytes in dark brown eyes have greater melanin content, which provides protection from ROS and reduces the risk of malignant transformation.^[^ ^19^ ^]^ Since Cdots induce ROS production, understanding the effects of elevated ROS on tumor growth is critical before the application of Cdots in cancer detection and targeted therapy. Here, we examine the effects of Cdot‐induced ROS on uveal melanoma cell viability, migration, invasiveness, and tumorigenicity as well as cell signaling and metabolomics.

## 2 Results and Discussion

### 2.1 Characterization of Cdots

**Figure 1:**
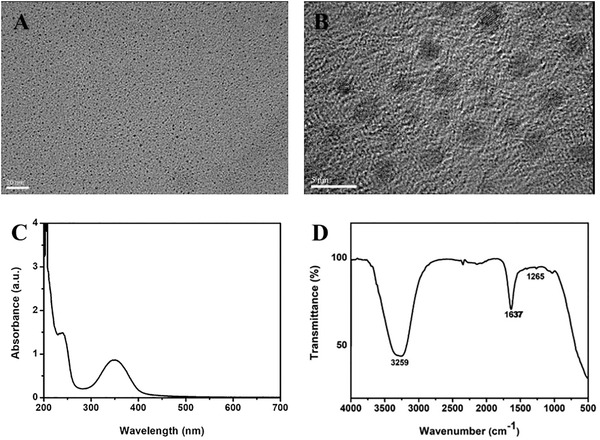
Cdot characterization. A,B) HRTEM images. Scale bars = 20 nm in panel (A) and 5 nm in panel (B). C) UV–vis absorbance spectrum. D) FTIR spectrum.

The Cdots were well dispersed, ≈3 nm in average diameter and exhibited a significant crystalline structure based on high‐resolution transmission electron microscopy (HRTEM) images (**Figure** **1**A,B). Cdot UV–vis spectra exhibited absorption peaks at 240 and 352 nm that were attributed to the *π*–*π*∗ transition of the conjugated C=C structure and the *n*–*π*∗ C=O transition, respectively (Figure 1C). Fourier transform infrared (FTIR) spectra showed peaks at 3259 cm^−1^ corresponding to vibrations of O—H and N—H bonds and at 1637 and 1265 cm^−1^ corresponding to plane bending vibrations of C in C=C and C—O—C bonds, indicating the functional groups —COOH, —OH, and —NH_2_ (Figure 1D).

### 2.2 Cdots Promote UM Cell Growth

Low cytotoxicity of Cdots is important for applications in UM detection and therapy. We assessed the viability of normal retinal pigment epithelium (RPE) cells and Pig1 melanocytes and two UM cell lines, Mum2B and 92.1, during a 72 h incubation with Cdots at 0, 25, 50, 100, and 200 µg mL^−1^. Exposure to Cdots at 0–200 µg mL^−1^ had no significant effect on the viability of normal RPE or Pig1 cells over the 72 h period (Figure S1A,B, Supporting Information). In contrast, exposure to Cdots at 200 µg mL^−1^ inhibited UM cell growth after 48 h (Figure S1C,D, Supporting Information).

**Figure 2:**
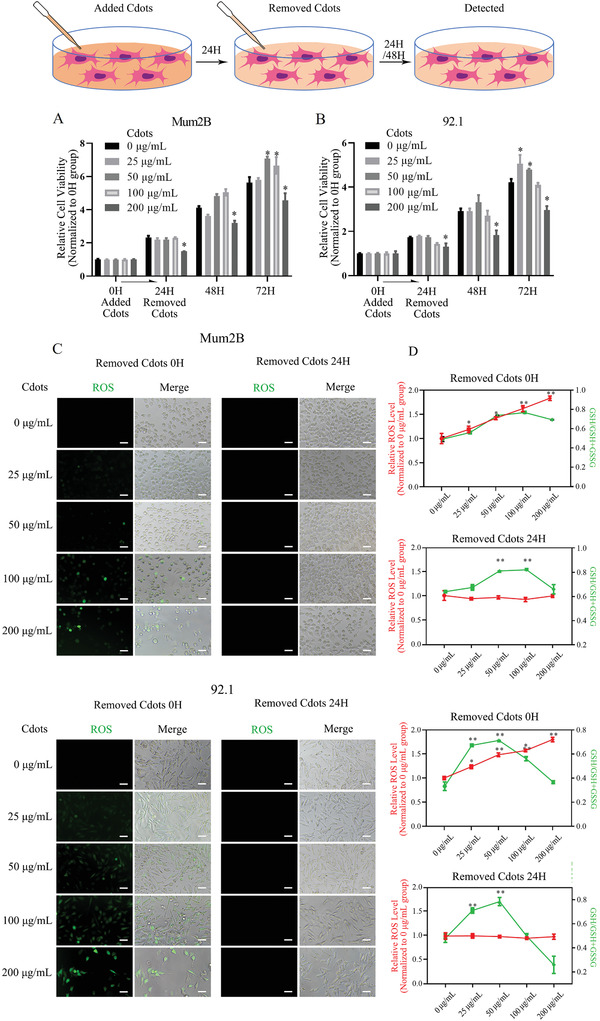
Cdots promote UM cell viability, ROS generation, and redox state balance. A,B) CCK‐8 assay results. Mum2B and 92.1 UM cells were cultured with Cdots for 24 h, Cdots were removed from the media, and the cells were cultured for another 48 h. After the 72 h period, Cdots at 50 and 100 µg mL^−1^ promoted Mum2B cell viability, and Cdots at 25 and 50 µg mL^−1^ promoted 92.1 cell viability. The results were compared to viability at time zero. C) Bright‐field and fluorescence images showing ROS levels in Cdot‐treated UM cells using a DCFH‐DA assay (*n* = 3 for each group). Scale bars = 50 µm. D) Quantification of ROS levels (red line) using a fluorometer showed that Cdots caused a concentration‐dependent increase in ROS after 24 h of culture. The ROS levels diminished after Cdots were removed for 48 h. A GSH/GSH + GSSG assay (green line) showed that Cdots increased the GSH/GSH + GSSG ratio at 50 and 100 µg mL^−1^ in Mum2B cells and at 25 and 50 µg mL^−1^ in 92.1 cells. The results were compared to those of the 0 µg mL^−1^ group. Asterisks indicate a statistically significant difference between the control and treatment groups. *n* = 3, *P < 0.05, **P < 0.01.

Nanomaterials injected into humans are typically removed from target tissues within 24 h.^[^ ^20^ ^]^ To simulate the long‐term (>24 h) effects of Cdots following removal from target tissues, we cultured normal and UM cells with Cdots at 0, 25, 50, 100, and 200 µg mL^−1^ for 24 h, removed the Cdots and continued culturing the cells for an additional 24 h, and counted the viable cells. Exposure of RPE and Pig1 cells to Cdots at various concentrations did not influence cell growth during the second 24 h period (Figure S2, Supporting Information). Surprisingly, exposure of Mum2B cells to Cdots at 50 and 100 µg mL^−1^ and of 92.1 cells to Cdots at 25 and 50 µg mL^−1^ promoted cell growth during the second 24 h period (**Figure** **2**A,B). At 200 µg mL^−1^, Cdots inhibited the growth of both UM cell types, as they underwent apoptosis after 24 h of exposure (Figure S3, Supporting Information). The fate of ROS as a signaling molecule or toxic agent depends on the concentration of ROS and the abundance of antioxidants.^[^ ^21^ ^]^ Compared with normal cells, UM cells are more sensitive to the influence of ROS. Therefore, high ROS levels can induce oxidative damage and UM cell death.

### 2.3 Cdots Restore Redox Balance in UM Cells by Inducing ROS

Redox balance is critical for cell growth and ROS play an important role in regulating the redox state. Moderate levels of ROS may activate proliferation‐related signaling pathways and promote tumor cell growth.^[^ ^22^ ^]^ Considering that Cdots possess oxygen‐containing groups, including carboxy and hydroxy groups, endocytosed Cdots may induce intracellular ROS accumulation. Hence, we tested cellular ROS levels after treatment with Cdots. UM cells were exposed to Cdots at 0, 25, 50, 100, and 200 µg mL^−1^ for 24 h, and the ROS level was assessed by dichlorodihydrofluorescein diacetate (DCFH‐DA) fluorescence staining (Figure 2C) and quantified using a fluorescence microplate reader (Figure 2D, red line). Both Mum2B and 92.1 cells exhibited a Cdot‐dose‐dependent increase in ROS. After removing the Cdots and continuing cell culture for an additional 24 h, the ROS levels of all Cdot‐treated groups returned to the level of the untreated control group. These results indicated that intracellular ROS levels were directly related to the Cdot concentration.

ROS can be scavenged by reduced glutathione (GSH), the active form of a tripeptide composed of glutamic acid, glycine, and cysteine, which acts as an intracellular antioxidant that protects cells during proliferation and differentiation.^[^ ^23^ ^]^ GSH provides the most rapid metabolic response when cells are in a state of oxidative stress.^[^ ^24^ ^]^ When ROS levels are elevated, peroxidase catalyzes a reaction between GSH and H_2_O_2_ that converts GSH to oxidized glutathione (GSSG). The ratio of GSH to GSH + GSSG is commonly used as an indicator of redox state balance. In both UM cell lines, the GSH/GSH + GSSG ratio increased in the Cdot treatment groups relative to the untreated control group at Cdot concentrations of 25–100 µg mL^−1^ after 24 h of culture. The GSH/GSH + GSSG ratio remained high even after the Cdots were removed and culture was continued for another 24 h (Figure 2D, green line). These results indicated that exposure of UM cells to Cdots at moderate concentrations increased the GSH/GSH + GSSG ratio, consistent with the previously observed increase in intracellular ROS.

GSH plays an important role in promoting cancer cell growth, chemotherapy resistance, and tumorigenesis. In breast cancer cells, GSH expression is increased by PI3K signaling.^[^ ^25^ ^]^ In neuroblastoma and Hodgkin's lymphoma, the oncogenes n‐myc and c‐myc enhance amino acid transport and promote GSH synthesis. Here, Cdots at a moderate concentration (50 µg mL^−1^) induced ROS production in UM cells, resulting in increased synthesis of GSH to scavenge ROS, restore redox balance, and support UM cell growth. Conversely, Cdots at a higher concentration (200 µg mL^−1^) inhibited UM cell growth and caused UM cell apoptosis due to overwhelming ROS levels.

### 2.4 Cdots Promote UM Cell Growth, Migration, and Invasiveness by Inducing ROS

**Figure 3:**
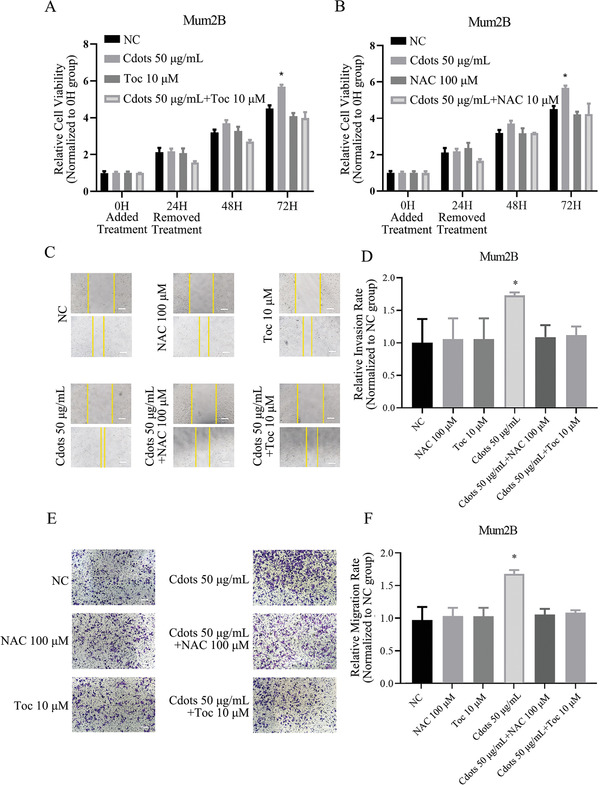
Cdots promote UM cell viability, migration, and invasiveness by inducing ROS. A,B) Addition of the antioxidants Toc and NAC reversed the Cdot‐induced proviability effect in Mum2B cells. The results were compared to those of the NC group. C) Microscopy images of Mum2B cells treated with Cdots with or without antioxidants in a cell scratch migration assay. Scale bars = 100 µm. D) Quantitation of Mum2B cell migration distance from the images presented in panel (C). Addition of the antioxidants reversed the Cdot‐induced promigration effect. The results were compared to those of the NC group. E) Microscopy images of Mum2B cells treated with Cdots with or without antioxidants in a Transwell cell invasion assay. F) Quantitation of the cell invasion images shown in panel (E). The addition of antioxidants reversed the Cdot‐induced proinvasion effect. The results were compared to those of the NC group. Asterisks indicate a statistically significant difference between the control and treatment groups. *n* = 3, *P < 0.05. Scale bars = 100 µm.

To further examine the effects of Cdot‐induced ROS on UM cell phenotypes, we compared UM cell growth, migration, and invasiveness using the following treatment groups: treatment with a combination of Cdots (50 µg mL^−1^) and ROS inhibitor (*N*‐acetyl‐l‐cysteine (NAC) at 100 µm, or *α*‐tocopherol (Toc) at 10 µm); treatment with Cdots alone; treatment with ROS inhibitor alone; and an untreated control group. Whereas Cdots alone at 50 µg mL^−1^ caused a significant increase in Mum2B cell growth after 72 h of culture, Cdots plus antioxidant (either NAC or Toc) prevented this effect, resulting in a cell proliferation rate similar to those of the untreated group or of Mum2B cells treated with antioxidant only (**Figure** **3**A,B). This result showed that the proviability effect of Cdots on UM cells was mediated by induced ROS.

Similarly, whereas Cdots at 50 µg mL^−1^ significantly promoted Mum2B cell migration and invasion, Cdots plus antioxidant (either NAC or Toc) prevented these effects, resulting in cell migration and invasion similar to those observed in untreated UM cells or in UM cells treated only with antioxidant (Figure 3C–F). These results demonstrated that the promigration and proinvasion effects of Cdots on UM cells were mediated by induced ROS. Cdots at a higher concentration (200 µg mL^−1^) had no significant effect on UM cell migration and a negative influence on invasion (Figures S4 and S5, Supporting Information).

### 2.5 Cdots Promote UM Cell Tumorigenicity

**Figure 4:**
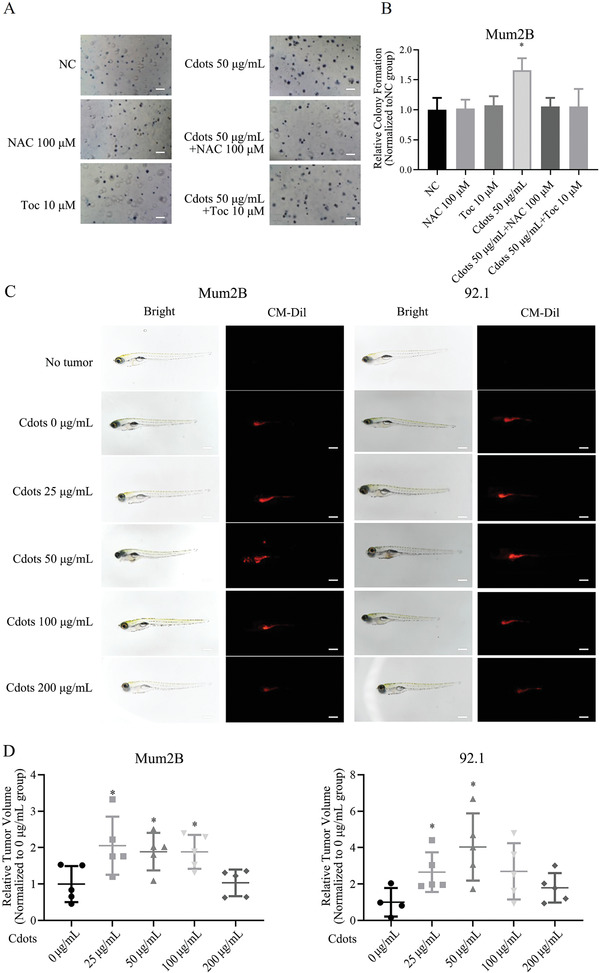
Exposure of UM cells to Cdots promotes tumorigenesis by inducing ROS. A) Images of UM cells exposed to Cdots and/or the antioxidants NAC or Toc. B) Quantitation of colony number. The addition of the antioxidants NAC or Toc reversed the Cdot‐induced procolony formation effect. The results were compared to those of the NC group. *n* = 3. C) Bright‐field and fluorescence images of zebrafish transplanted with UM cells labeled with CM‐Dil dye and exposed to Cdots at different concentrations (*n* = 5 for each group). D) Quantitation of tumor volume in fluorescence images. Cdots at 25, 50, and 100 µg mL^−1^ promoted Mum2B cell tumorigenicity and Cdots at 25 and 50 µg mL^−1^ promoted 92.1 cell tumorigenicity. The results were compared to those of the 0 µg mL^−1^ group. Asterisks indicate a statistically significant difference between the control and treatment groups. *n* = 5, *P < 0.05. Scale bars = 100 µm.

We next investigated the effects of Cdot‐induced ROS on UM cell tumorigenesis using an in vitro colony formation assay, a zebrafish xenograft model, a nude mouse subcutaneous xenograft model, and an intraocular xenograft model. Whereas Cdots at 50 µg mL^−1^ significantly promoted Mum2B cell colony formation, cotreatment with Cdots at 50 µg mL^−1^ and NAC at 100 × 10^−6^ m or Toc at 10 × 10^−6^ m eliminated this effect, resulting in a number of colonies similar to those of untreated Mum2B cells or cells treated with antioxidant alone (**Figure** **4**A,B). This result showed that the protumorigenic effect of Cdots on UM cells in vitro was mediated by induced ROS. Cdots at a higher concentration (200 µg mL^−1^) inhibited UM cell tumorigenicity (Figure S6, Supporting Information).

**Figure 5:**
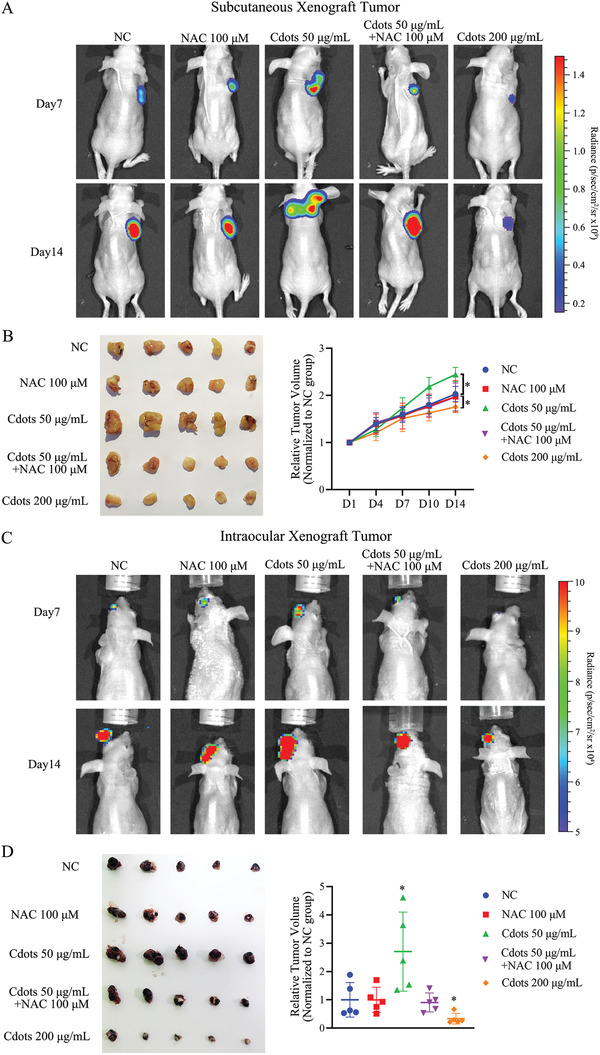
Exposure of UM cells to Cdots promotes tumorigenesis by inducing ROS in both nude mouse subcutaneous and intraocular xenograft models. A) Luciferase images of tumor‐bearing mice at various time points after subcutaneous injections of pretreated UM cells. Addition of the antioxidant NAC reversed the Cdot‐induced tumorigenic effect. Cdots at 200 µg mL^−1^ inhibited tumor growth. B) Tumors were compared at the end of the experiment. Tumor growth curves were measured starting at 1 d after inoculation (*n* = 5 for each group). The results were compared to those of the NC group. C) Luciferase images of tumor‐bearing mice at various time points after intraocular injection of treated mouse melanoma cells. Addition of the antioxidant NAC reversed the Cdot‐induced protumorigenesis effect. Cdots at 200 µg mL^−1^ inhibited tumor growth. D) Comparison of tumor size at the end of the experiment. Tumor growth was monitored starting at 1 d after inoculation (*n* = 5 for each group). The results were compared to those of the NC group. Asterisks indicate a statistically significant difference between the control and treatment groups. *n* = 5, *P < 0.05.

In the zebrafish xenograft model, UM Mum2B and 92.1 cells were labeled with the red fluorescent dye CM‐Dil and injected into the zebrafish yolk sac. The zebrafish were cultured in water containing Cdots for 24 h and then without Cdots for 7 d. Tumor size was measured using fluorescence microscopy. Cdots at 25, 50, and 100 µg mL^−1^ promoted significantly greater Mum2B tumor growth than no treatment and Cdots at 25 and 50 µg mL^−1^ promoted 92.1 cell tumor growth (Figure 4C,D). In the nude mouse subcutaneous xenograft model, Mum2B cells were pretreated with negative control (NC) or NAC at 100 × 10^−6^ m, Cdots at 50 µg mL^−1^, Cdots at 50 µg mL^−1^ together with NAC at 100 × 10^−6^ m, and Cdots at 200 µg mL^−1^ for 24 h. Then, the pretreated Mum2B cells suspended in 100 µL Basement Matrigel were subcutaneously implanted in the flanks of nude mice. Whereas Cdots at 50 µg mL^−1^ significantly promoted tumor growth, cotreatment with Cdots at 50 µg mL^−1^ and NAC at 100 × 10^−6^ m eliminated this effect, resulting in tumor growth similar to that of untreated cells or cells treated with antioxidant alone. Cdots at a higher concentration (200 µg mL^−1^) significantly inhibited UM cell tumorigenicity (**Figure** **5**A,B). To validate these findings in another tumor model, B16F10 cells were pretreated in the same manner as in the subcutaneous xenograft model and directly injected into the left eye of nude mice to establish an intraocular xenograft model.^[^ ^26^ ^]^ The trend of tumor growth observed in this model was consistent with that in the subcutaneous xenograft model (Figure 5C,D). These results showed that the tumorigenic effect of Cdots on UM cells in vivo was mediated by induced ROS.

### 2.6 Cdot‐Induced ROS Increase Amino Acid and Fatty Acid Metabolism

Carbon nanomaterials have been shown to induce ROS and cause DNA denaturation with carcinogenic effects. For example, carbon nanotubes cause DNA damage and nuclear particle formation in human bronchial epithelial cells. Graphene oxide causes DNA fragmentation and chromosome instability in human mesenchymal stem cells and neonatal fibroblasts.^[^ ^27^ ^]^ Carbon nanodiamonds increase the expression of markers of DNA fragmentation (p53, OGG‐1, Rad51, and XRCC‐4) in mouse embryonic cells, indicating an increased risk of tumor initiation.^[^ ^28^ ^]^ Newly developed graphene quantum dots increase the expression of the DNA damage‐related genes p53, Rad51, and OGG1.^[^ ^29^ ^]^ Respiratory exposure to carbon nanotubes can stimulate the MAPK, NF‐*κ*B, and Akt signaling pathways, promoting the development of lung cancer.^[^ ^30^ ^]^ Since metabolic changes precede the activation of signaling pathways and DNA damage, we assessed metabolic changes in Cdot‐treated UM cells to gain insight into the mechanisms underlying the protumorigenic effects of Cdots.

**Figure 6:**
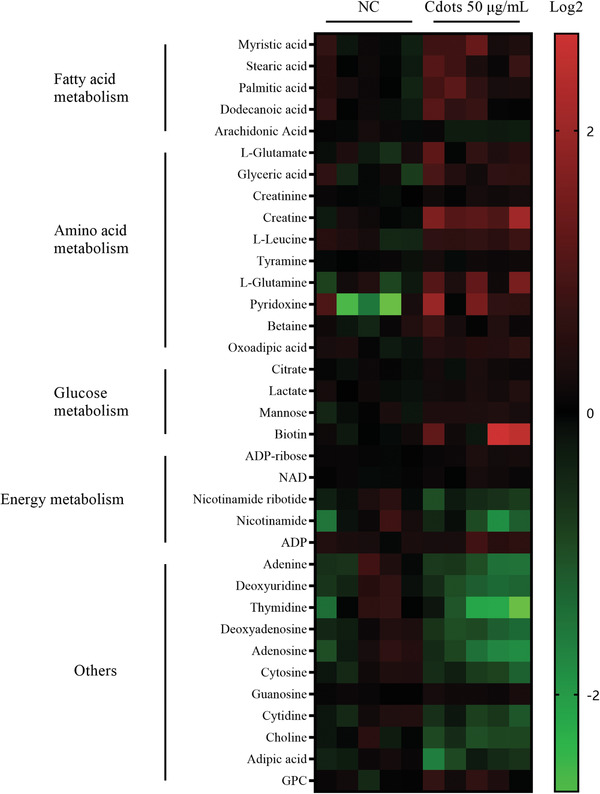
Cdot‐induced ROS increase amino acid and fatty acid metabolism. Heatmap depicting changes in metabolite concentration between control and 50 µg mL^−1^ Cdot‐treated Mum2B cells (*p* < 0.05). Intracellular metabolites were analyzed by LC‐MS. Red indicates increased metabolite abundance and green indicates decreased metabolite abundance.

We used liquid chromatograph‐mass spectrometer (LC‐MS) to measure changes in metabolites in Mum2B cells after exposure to 50 µg mL^−1^ Cdots for 24 h compared with those in untreated control cells (**Figure** **6**). The Cdot treatment group exhibited greater synthesis and metabolism of amino acids, including glutamate (Glu), glutamine (Gln), leucine (Leu), tyrosine (Tyr), pyridoxine (amino acid transaminase and decarboxylase coenzyme), and biotin (amino acid carboxylase and decarboxylase coenzyme), and amino acid metabolites, including glycolic acid, betaine, creatine, and creatinine. The level of saturated fatty acids was higher in the Cdot treatment group, including higher levels of lauric acid, myristic acid, palmitic acid, and stearic acid; the level of the unsaturated fatty acid arachidonic acid was lower in the Cdot treatment group.

### 2.7 Cdot‐Induced ROS Activate Mammalian Target of Rapamycin (mTOR) Signaling and Enhance Glutamine Metabolism

Tumor cells alter their metabolism to meet the needs of rapid proliferation.^[^ ^31^ ^]^ For example, tumor cells are highly dependent on glutamine, which provides nitrogen and carbon for the synthesis of amino acids, fatty acids, and nucleic acids, to supplement the intermediate products needed by the tricarboxylic acid cycle and to meet the needs of vigorous proliferation and division.^[^ ^32^ ^]^ Whereas normal cells prefer to uptake exogenous fatty acids, tumor cells synthesize fatty acids de novo for biosynthesis of membranes and signaling molecules.^[^ ^33^ ^]^ Tumor cells also commonly decouple glycolysis from pyruvate oxidation and the tricarboxylic acid cycle (the Warburg effect) for quicker energy production.^[^ ^34^ ^]^ Due to the importance of glutamine and its significant change after exposure to Cdots, we focused on changes in glutamine metabolism‐related gene expression in Cdot‐treated UM cells.

**Figure 7:**
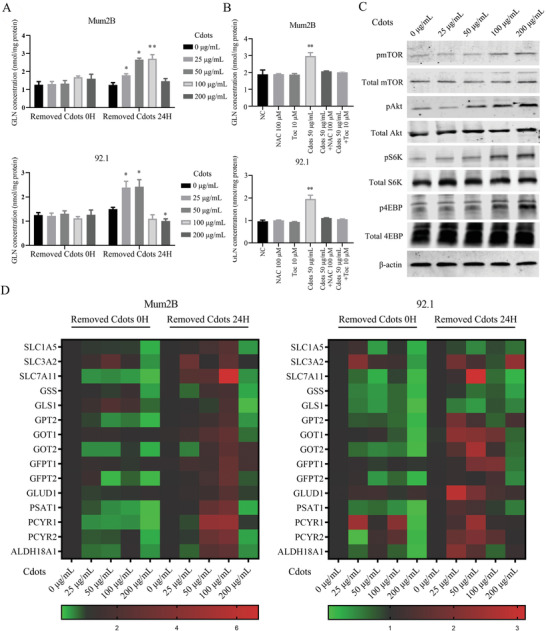
Cdot‐induced ROS activate mTOR signaling and increase the expression of downstream glutamine metabolism‐related genes. A) UM intracellular glutamine content increased in a Cdot‐concentration‐dependent manner after removal of Cdots and 24 h of additional culture. B) Addition of the antioxidants NAC and Toc reversed the Cdot‐induced upregulation of intracellular glutamine concentration. C) Western blots showing that pAkt, pmTOR, pS6K, and p4EBP protein levels increased in a Cdot‐concentration‐dependent manner after removal of Cdots and 24 h of additional culture. Total Akt, total mTOR, total S6K, total 4EBP, and *β*‐actin were used as internal references. D) The expression levels of glutamine metabolism‐related genes increased when Mum2B cells were exposed to Cdots at 50 and 100 µg mL^−1^ and when 92.1 cells were exposed to Cdots at 25 and 50 µg mL^−1^. The data represent averages from three independent experiments and are normalized to 18S. The asterisks indicate a statistically significant difference between the control and treatment groups. *n* = 3, *P < 0.05, **P < 0.01.

A glutamine detection kit was used to verify the LC‐MS results. There were no significant changes in intracellular glutamine levels in Mum2B or 92.1 cells cultured with different concentrations of Cdots for 24 h (**Figure** **7**A). However, after removal of Cdots and an additional 24 h of culture, increases in glutamine levels were observed in Mum2B cells exposed to 50 and 100 µg mL^−1^ Cdots and in 92.1 cells exposed to 25 and 50 µg mL^−1^ Cdots. Furthermore, Cdots at 50 µg mL^−1^ significantly elevated the glutamine level, whereas cotreatment with Cdots at 50 µg mL^−1^ and NAC at 100 × 10^−6^ m or Toc at 10 × 10^−6^ m abrogated this effect, resulting in a level of glutamine similar to that of untreated Mum2B cells or cells treated with antioxidant alone. These results showed that the Cdot‐induced upregulation of glutamine levels was mediated by ROS induction in UM cells (Figure 7B).

mTOR is an important intracellular sensor and metabolism regulator in tumor cells. mTOR forms two multiprotein complexes, mTORC1 and mTORC2. mTORC1 is regulated by the Akt signaling pathway. Akt blocks tuberous sclerosis complex 2 (TSC2) and activates mTORC1 by phosphorylation of Rheb GAPs.^[^ ^35^ ^]^ Akt has been associated with enhanced oxygen consumption and ROS production in cells under glucose starvation conditions,^[^ ^36^ ^]^ indicating that the Akt/mTORC1 signaling pathway helps regulate the oxidative balance in tumor cells. mTORC1 also mediates metabolic reprogramming by directly activating ribosomal protein S6 kinase (S6K) and inhibiting eIF4E‐binding protein (4EBP) to increase the translation of metabolic enzymes and metabolism‐related transcription factors.^[^ ^37^ ^]^ In amino acid metabolism, mTORC1 signaling has been shown to increase the expression of glutamine and glutamate dehydrogenase, accelerate the decomposition of glutamine to glutamate and *α*‐ketoglutarate, increase the expression of ornithine decarboxylase and argininosuccinate synthetase, promote the metabolism of arginine, increase the expression of SLC7A5 and SLC43A1, and enhance the uptake of branched chain amino acids.^[^ ^38^ ^]^ In fatty acid metabolism, mTORC1 signaling increases the expression of fatty acid synthetase and stearoyl CoA dehydrogenase 1 (SCD1) and accelerates fatty acid synthesis.^[^ ^39^ ^]^

We used Western blots to investigate whether the Akt/mTOR signaling pathway participated in the metabolic changes caused by Cdot‐induced ROS in UM cells. The expression of phosphorylated Akt, mTOR, S6K and 4EBP increased with increasing Cdot concentration (Figure 7C), indicating the activation of the Akt/mTOR signaling pathway by Cdot‐induced ROS.

We used quantitative real‐time polymerase chain reaction (qRT‐PCR) to analyze the changes in the glutamine metabolism‐related genes *SLC7A11*, *SCL3A2*, *PYCR1*, *PYCR2*, *PSAT1*, *GPT2*, *GOT1*, *GOT2*, *GLUD1*, *GFPT1*, *GFPT2*, and *ALDH18A1* in Cdot‐treated UM Mum2B and 92.1 cells. There were no significant changes in the mRNA expression of these glutamine metabolism‐related genes in Mum2B or 92.1 cells cultured with different concentrations of Cdots for 24 h (Figure 7D). However, after the removal of Cdots and an additional 24 h of culture, the mRNA expression of these genes was significantly increased in Mum2B cells exposed to Cdots at 50 and 100 µg mL^−1^ and in 92.1 cells exposed to Cdots at 25 and 50 µg mL^−1^. In contrast, the mRNA expression of these genes decreased significantly when either UM cell type was exposed to Cdots at 200 µg mL^−1^. These results indicated that Cdot‐induced ROS upregulate the expression of glutamine metabolism‐related genes in uveal melanoma cells. Increased glutamine metabolism could accelerate the transformation of glutamine into glutamate and glutamate into *α*‐ketoglutarate, which enters the tricarboxylic acid cycle and generates adenosine triphosphate (ATP), promoting tumor cell growth.

**Figure 8:**
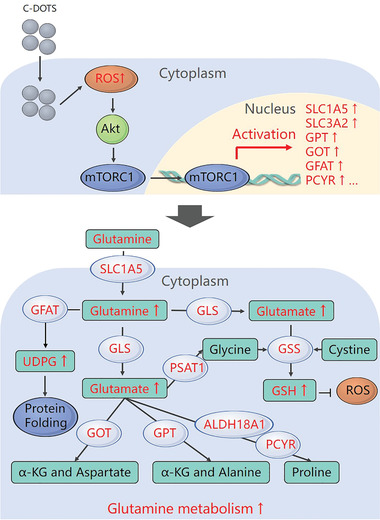
Schematic of the effects of Cdots on glutamine metabolism in tumor cells.

Based on these results, we propose a potential mechanism by which Cdots stimulate UM cell development (shown in **Figure** **8**). After Cdots are endocytosed, intracellular ROS are elevated. Moderate ROS act as stimulating signaling molecules that activate Akt/mTOR signaling, resulting in upregulated glutamine‐related gene expression and accelerated glutamine metabolism, which promotes UM cell growth. High concentrations of Cdots stimulate excess ROS production, which leads to UM cell death.

## 3 Conclusion

This study demonstrates that moderate Cdot concentrations promote UM cell tumorigenesis, as demonstrated by the reversal of this effect with the addition of antioxidants. The protumorigenic effect was mainly due to the Cdot‐induced production of ROS, which activated Akt/mTOR signaling and increased glutamine metabolism, thus promoting UM cell proliferation and metastasis. Hence, the concentration of Cdots used in cancer detection and therapeutic applications must be carefully investigated.

## 4 Experimental Section

##### Cdot Synthesis and Characterization

Cdots were synthesized using a modified hydrothermal method described previously.^[^ ^40^ ^]^ In a typical experiment, 2 g of citrate (Sigma‐Aldrich, USA) and 1 g of l‐tryptophan (L‐Trp) (Sigma‐Aldrich, USA) were dissolved in 30 mL of deionized water and stirred for 1 h to form a homogeneous solution. The solution was heated at 160 °C for 2 h in a polytetrafluoroethylene (Teflon)‐lined autoclave reactor (Autoclave, USA), and the resulting homogeneous dark brown solution was allowed to cool to room temperature. The solution was centrifuged at 12 000 rpm for 10 min to remove unreacted precipitates. Excess citric acid and L‐Trp were removed by repeated dialysis (1000 Da cutoff) against deionized water for 2 d. Dry Cdots were collected by freeze‐drying and weighed and dissolved in phosphate‐buffered saline (PBS) for further use. Cdot size and morphology were characterized by using a JEM‐2100 transmission electron microscope (JEOL, Japan). Cdot UV–vis absorption spectra were collected using a Varian Cary 50 UV–vis Spectrophotometer (Varian, Inc., USA). Cdot FTIR spectra were collected using a Nicolet 6700 FTIR spectrometer (Thermo Electron Corporation, USA).

##### Cell Culture

Cell culture was performed as described previously.^[^ ^41^ ^]^ The human normal RPE cell line, human normal melanocyte cell line (Pig1), and mouse melanoma cell line (B16F10) were purchased from the Cell Bank of the Chinese Academy of Sciences, Shanghai, China. The human UM cell lines Mum2B and 92.1 were kindly provided by J. F. Marshall (Tumour Biology Laboratory, John Vane Science Centre, London, UK). Briefly, Mum2B, 92.1, B16F10, RPE, and Pig1 cells were grown in Roswell Park Memorial Institute 1640 (RPM 1640) complete medium supplemented with 10% fetal bovine serum (FBS; Gibco, USA) and 1% penicillin‐streptomycin (Gibco, USA) at 37 °C and 5% CO_2_. For routine maintenance, cells were digested using trypsin‐ethylene diamine tetraacetic acid (EDTA) solution when cultures reached 80% confluency and were reseeded on tissue culture‐treated polystyrene plates (Corning, USA) at a split ratio of 1:3.

##### Analysis of UM Cell ROS Level and Redox State In Vitro

The effect of Cdots on UM cell ROS level was assessed using a DCFH‐DA assay. UM cells were seeded into a flat‐bottomed 96‐well culture plate at 2×10^3^ cells per well and incubated with Cdots at 0, 25, 50, 100, or 200 µg mL^−1^ for 24 h. The cell culture medium was removed, RPMI 1640 serum‐free medium containing 10 × 10^−6^ m DCFH‐DA (Beyotime, China) was added to the culture plate, and the cells were incubated at 37 °C and 5% CO_2_ for 20 min. The cells were washed three times with PBS, and images were acquired by fluorescence microscopy using 488 nm excitation and 525 nm emission filters. The effects of Cdots on the UM cell redox state were assessed using a GSH/GSH + GSSG Quantification Colorimetric Kit (BioVision, USA) and a Benchmark Microplate Reader (Bio‐Rad, USA).

##### Measurement of Cell Viability, Apoptosis, Migration, and Invasiveness In Vitro

The effects of Cdots on normal and UM cell viability were measured using a Cell Counting Kit‐8 (CCK‐8) colorimetric assay (Takara Bio, Japan).^[^ ^42^ ^]^ Cells were seeded into a flat‐bottomed 96‐well culture plate at 2×10^3^ cells per well and cultured for 3 h for cell attachment before incubation with Cdots at 0, 25, 50, 100, or 200 µg mL^−1^. For rescue experiments, cells were incubated with either negative control, NAC (100 × 10^−6^ m), *α*‐tocopherol (10 × 10^−6^ m), Cdots (50 µg mL^−1^), Cdots (50 µg mL^−1^) together with 100 × 10^−6^ m NAC (dissolved in 20 × 10^−3^ m PBS), or Cdots (50 µg mL^−1^) together with 10 × 10^−6^ m *α*‐tocopherol (dissolved in 2 × 10^−3^ m dimethyl sulfoxide (DMSO)). At 24 h, the media containing the different treatments were removed and replaced with fresh medium. At 0, 24, 48, or 72 h, 10 µL of CCK‐8 solution was added and the cells were incubated for 3 h at 37 °C and 5% CO_2_. The relative cell number in each sample was assessed based on formazan production by measuring absorbance at 450 nm using a Benchmark Microplate Reader. The effects of Cdots on UM cell apoptosis were measured using a Fluorescence activated Cell Sorting (FACS) assay. Cells were seeded into a flat‐bottomed six‐well culture plate at 5×10^5^ cells per well and incubated with Cdots at 0, 50, or 200 µg mL^−1^ for 24 h. Then, the cells were permeabilized with 70% ethanol at 4 °C for 30 min. After treatment with propidium iodide and Annexin V, cell apoptosis was detected by a FACSCanto flow cytometer (BD Biosciences, USA) and analyzed using ModFit LT V3.0 software (Verity Software House, USA). A cell scratch assay was used to assess the effects of Cdots on UM cell migration.^[^ ^43^ ^]^ Cells were seeded into a 24‐well culture plate at 1×10^5^ cells per well and cultured with Cdots at 0, 25, 50, 100, or 200 µg mL^−1^ until 80% confluence. For the rescue experiment, the same treatment groups used for the CCK‐8 assay described above were included. A 10 µL pipette tip was used to evenly scratch the culture plate. The cells were washed with PBS, and fresh medium was added. Images of the cells were acquired at time zero and at 24 h, and the cell migration distance was determined using ImageJ (Version 1.8.0). A Transwell assay was used to measure the effects of Cdots on UM cell invasion.^[^ ^44^ ^]^ Transwell chambers were purchased from BD Biosciences (USA). Briefly, UM cells were pretreated with Cdots at 0, 25, 50, 100, or 200 µg mL^−1^ for 24 h. For the rescue experiment, the setup of treatment was the same as that of the CCK‐8 assay. Then, the Cdots‐treated UM cells were digested, and 1×10^4^ cells were suspended in a Transwell chamber with two compartments; the upper and lower compartments contained RPMI 1640 medium with 2% and 10% FBS, respectively. After incubating the cells for 12 h at 37 °C and 5% CO_2_, the Transwell chamber was stained using 0.25% crystal violet (Solarbio, China). The cells on the inner side of the chamber were scrubbed by cotton swabs and the cells on the outer side were photographed. The number of invasive cells was determined using ImageJ.

##### UM Cell Tumorigenicity In Vitro and in a Zebrafish Xenograft Model

A colony formation assay was used to measure the effects of Cdots on UM cell tumorigenesis in vitro.^[^ ^45^ ^]^ Briefly, 250 µL of 0.6% agar (Sigma‐Aldrich, USA) complete medium was spread in each well of a 24‐well plate to obtain the bottom layer. UM cells were pretreated with Cdots at 0, 25, 50, 100, or 200 µg mL^−1^ for 24 h. For the rescue experiment, the treatment setup was the same as that of the CCK‐8 assay. Then, the Cdots‐treated UM cells were digested and 1×10^3^ cells were resuspended in 1.0 mL of 0.3% agar complete medium and seeded into the upper layer. The cells were cultured with 300 mL of complete medium for 2 weeks. The colonies in the soft agar were stained with 0.1% crystal violet (Solarbio, China) and imaged and the colony number was counted. A zebrafish xenograft model was used to assess the effects of Cdots on UM cell tumorigenesis in vivo. Fertilized zebrafish eggs were incubated for 24 h at 28 °C and then 1 mg mL^−1^ Pronase E solution (Roche, USA) was used to break fertilized egg membranes. Zebrafish embryos in good condition were selected for transplantation with UM cells. UM cells from the Mum2B and 92.1 cell lines were digested using trypsin‐EDTA solution, mixed with serum‐free RPMI 1640 medium containing 1 × 10^−6^ m CellTracker CM‐Dil dye (Thermo Fisher, USA), and incubated at 37 °C and 5% CO_2_ for 5 min. After labeling, the cell precipitate was washed three times with PBS, and the cells were resuspended in serum‐free RPMI 1640 medium at 3 × 10^7^ cells mL^−1^. Under a stereomicroscope, 10 nL of the UM cell suspension was injected into the posterior part of the yolk sac of zebrafish embryos under anesthesia using tricaine methanesulfonate. The zebrafish embryos were then incubated with Cdots at different concentrations. After 24 h of culture at 28 °C, dead zebrafish were removed, and fresh zebrafish embryo water was added. After 7 d, the juvenile fish were anesthetized and imaged using an inverted fluorescence microscope. ImageJ was used to analyze the optical density of tumor cells. All procedures performed in studies involving animals were in accordance with the ethical standards of the Ethics Committee of the Institutional Ethical Review Board of Shanghai Ninth People's Hospital.

##### Luciferase‐Expressing UM Cell Establishment and UM Cell Tumorigenicity in a Nude Mouse Xenograft Model

The luciferase sequence was cloned into the pLVX‐mCherry vector (Addgene, USA). Transduction and viral infection were performed as previously described.^[^ ^46^ ^]^ Subsequently, luciferase‐expressing Mum2B and B16F10 cells were incubated with negative control, 100 × 10^−6^ m NAC, 50 µg mL^−1^ Cdots, 50 µg mL^−1^ Cdots together with 100 × 10^−6^ m NAC, or 200 µg mL^−1^ Cdots for 24 h. For the subcutaneous xenograft model, the pretreated Mum2B cells were suspended in 100 µL of Basement Matrigel (BD Biosciences, USA) and injected into the subcutaneous tissue of BALB/c nude mice. For the intraocular xenograft model, pretreated B16F10 cells were directly injected into the left eye of BALB/c nude mice. The tumor volume was recorded twice weekly. The animals were imaged using a VivoVision Systems Lumazone imaging system (Mag Biosystems, Tucson, AZ, USA) at days 7 and 14 postinjection. The animals were sacrificed at day 14 and the tumor volume was calculated by the following formula: tumor volume = *π*/6(s1 × s2 × s2), where s1 was the largest tumor diameter and s2 was the smallest tumor diameter.

##### LC‐MS Analysis of UM Cell Metabolomics In Vitro

UM cells (1×10^7^) were seeded into a flat‐bottomed 10 cm culture plate and incubated with Cdots at 0 and 50 µg mL^−1^. At 24 h, media containing Cdots at different concentrations were removed and replaced with fresh medium. At 48 h, Mum2B cells were washed using ice‐cold PBS and mixed with a 2:2:1 mixture of acetonitrile, methanol, and 0.5 m formic acid (Sigma‐Aldrich, USA). The cells were scraped and collected in a clean centrifuge tube, and the samples were ultrasonicated on ice (30 cycles of 2 s on, 1 s off), frozen with liquid nitrogen and dried under a stream of nitrogen. The samples were then dissolved in 400 µL of ice‐cold ultrapure water and centrifuged at 20 000 g at 4 °C for 10 min. The supernatant was transferred to a new centrifuge tube, and the sample was diluted with 100 µL of high performance liquid chromatography (HPLC) solvent (50 µL glacial acetic acid, 450 mL ultrapure water, 180 µL thiobarbituric acid (TBA), pH 9.2) (Sigma‐Aldrich, USA). LC‐MS analysis was performed using an Agilent 1290 ultrahigh pressure LC system and an Agilent 6540 quadrupole time‐of‐flight mass spectrometer (Agilent, USA) using both positive and negative ion modes. The original data were transformed using Agilent MassHunter Qualitative Analysis software; peak recognition, time correction, automatic integration, and internal standard normalization were performed using the Bioconductor xcms package. Partial least squares discriminant analysis was used to identify metabolites (variable importance in projection (VIP) > 1, P < 0.05). Molecular weights and secondary mass spectra were also compared with the human metabolome database (HMDB) online database to identify metabolites.

##### Analysis of Glutamine Metabolism in UM Cells In Vitro

UM cells were pretreated with Cdots at 0, 25, 50, 100, or 200 µg mL^−1^ for 24 h before removal of Cdots. For the rescue experiment, the setup of the treatment was the same as that of the CCK‐8 assay. A Glutamine Colorimetric Assay Kit (BioVision, USA) was used to measure the effects of Cdots on UM intracellular glutamine content. qRT‐PCR was used to measure the expression of glutamine metabolism‐related genes. Total RNA was extracted using an RNeasy Mini Kit (Qiagen, Germany). A TaKaRa PrimeScript RT Reagent Kit (Takara Bio, Japan) was used to transcribe cDNA for qRT‐PCR analysis. Relative gene expression was measured using SYBR Green qPCR Master Mix (Applied Biosystems, USA) and an Applied Biosystems QuantStudio 6 Flex Thermal Cycler and normalized to the averaged expression of 18S mRNA for the following genes: *SLC7A11* (solute carrier family 7 member 11), *SLC1A5* (solute carrier family 1 member 5), *SLC3A2* (solute carrier family 3 member 2), *PCYR1* and *PCYR2* (pyrroline‐5‐carboxylate reductase 1 and 2), *PSAT1* (phosphoserine aminotransferase 1), *GSS* (glutamine synthetase), *GPT2* (glutamic pyruvic transaminase 2), *GOT1* and *GOT2* (glutamic oxaloacetic transaminase 1 and 2), *GLUD1* (glutamate dehydrogenase 1), *GLS1* (glutaminase 1), *GFPT1* and *GFPT2* (glutamine‐fructose‐6‐phosphate aminotransferase 1 and 2), and *ALDH18A1* (aldehyde dehydrogenase 18 family member A1). The primers used in qRT‐PCR are listed in **Table** **1**. The data were analyzed using Applied Biosystems QuantStudio Real‐Time PCR Software, and changes in expression were calculated using the ΔΔCT method. Relative mRNA expression levels were normalized against 18S.

**Table 1:** Primers used in qRT‐PCR

Gene	Forward (5'–3')	Reverse (5'–3')
*SLC7A11*	TCTCCAAAGGAGGTTACCTGC	AGACTCCCCTCAGTAAAGTGAC
*SLC1A5*	TCATGTGGTACGCCCCTGT	GCGGGCAAAGAGTAAACCCA
*SCL3A2*	TGAATGAGTTAGAGCCCGAGA	GTCTTCCGCCACCTTGATCTT
*PYCR2*	CAGCAACAAGGAGACGGTGA	CGTACACTGTAGCGCCTTCC
*PYCR1*	TGGCTGCCCACAAGATAATGG	CGTGACGGCATCAATCAGGT
*PSAT1*	TGCCGCACTCAGTGTTGTTAG	GCAATTCCCGCACAAGATTCT
*GSS*	GGGAGCCTCTTGCAGGATAAA	GAATGGGGCATAGCTCACCAC
*GPT2*	GTGATGGCACTATGCACCTAC	TTCACGGATGCAGTTGACACC
*GPT*	CTCTTGCCTGGAGTTCCCTCT	GAGGCCATGACTCTACCCAG
*GOT2*	AGCCTTACGTTCTGCCTAGC	AAACCGGCCACTCTTCAAGAC
*GOT1*	ATTTCTTAGCGCGTTGGTACA	ACACAGCATTGTGATTCTCCC
*GLUD1*	CGGGGAGTCTGAGAAAGCG	TAGCGGTACATGGCCACAAG
*GLS2*	GCCTGGGTGATTTGCTCTTTT	CCTTTAGTGCAGTGGTGAACTT
*GLS*	AGGGTCTGTTACCTAGCTTGG	ACGTTCGCAATCCTGTAGATTT
*GFPT2*	CCAACAGCAGGGATGCTACA	AGCACTTGGGTAGAAGGCAC
*GFPT1*	GGAATAGCTCATACCCGTTGG	TCGAAGTCATAGCCTTTGCTTT
*ALDH18A1*	GCCCTTCAACCAACATCTTCT	AGGGGTACAGTGATAAACGGG

##### Analysis of Akt/mTOR Signaling in UM Cells

Akt/mTOR signaling was assessed by Western blot. Radio‐immunoprecipitation assay (RIPA) lysis buffer (Beyotime, China) containing 1 × 10^−9^ m phenylmethylsulfonyl fluoride (PMSF) (Invitrogen, USA) was used to lyse cells, and then the collected protein was measured using a bicinchoninic acid protein assay kit (Thermo Fisher Scientific Inc., USA). Proteins were separated using 10% sodium dodecyl sulfate‐polyacrylamide gel (SDS‐PAGE) electrophoresis and transferred onto polyvinylidene pluoride (PVDF) membranes (Millipore, USA). Then, the membranes were incubated overnight at 4 °C with anti‐Phospho‐mTOR (clone 7C10, Cat. No. 2983, Cell Signaling Technology, USA), anti‐mTOR (clone D9C2, Cat. No. 5536, Cell Signaling Technology, USA), anti‐Phospho‐Akt (clone D9E, Cat. No. 4060, Cell Signaling Technology, USA), anti‐Akt (clone C67E7, Cat. No. 4691, Cell Signaling Technology, USA), anti‐Phospho‐p70 S6 Kinase (clone 108D2, Cat. No. 9234, Cell Signaling Technology, USA), anti‐p70 S6 Kinase (clone 49D7, Cat. No. 2704, Cell Signaling Technology, USA), and anti‐*β*‐actin (clone AC‐15, Cat. No. A5441, Sigma‐Aldrich, USA) antibodies. Anti‐mouse (1:5000) or anti‐rabbit (1:5000) fluorescein‐conjugated secondary antibodies (Abcam, USA) were used to detect immunoreactive bands after 1 h of incubation. Finally, the bands were visualized using Odyssey V3.0 image scanning (LI‐COR Biosciences, USA). Each protein was tested three times. Total Akt, total mTOR, total S6K, total 4EBP, and *β*‐actin were used as internal references.

##### Statistical Analysis

All of the in vitro experiments were performed in triplicate. All of the in vivo experiments were performed five times and the data are expressed as the mean ± standard deviation. Statistical analyses were performed in Statistical Product and Service Solutions (SPSS) 23.0 statistical software (IBM, USA). The differences between two groups were analyzed with unpaired two‐sided Student's *t*‐test. A *P*‐value < 0.05 was considered statistically significant and is indicated with asterisks, as described in the figure legends.

## Conflict of Interest

The authors declare no conflict of interest.

## Supporting information

Supporting Information

## Data Availability

The data that support the findings of this study are available from the corresponding author upon reasonable request.
