# Supplementary material for: Dose‐Dependent Carbon‐Dot‐Induced ROS Promote Uveal Melanoma Cell Tumorigenicity via Activation of mTOR Signaling and Glutamine Metabolism
Source: Adv Sci (Weinh). 2021 Feb 25;8(8):2002404. doi: 10.1002/advs.202002404 (PMC8061404; doi:10.1002/advs.202002404)
Supplement: Supplementary file 1 — Supporting Information [file ADVS-8-2002404-s001.pdf]

© 2021 Wiley-VCH GmbH

ADVANCED SCIENCE
Open Access

Supporting Information

for *Adv. Sci.,* DOI: 10.1002/advs.202002404

Dose-dependent Carbon Dot-induced ROS Promote Uveal Melanoma Cell Tumorigenicity via Activation of mTOR Signaling and Glutamine Metabolism

Yi Ding, Jie Yu, Xingyu Chen, Shaoyun Wang, Huixue Wang, Guangxia Shen, Renbing Jia, Shengfang Ge, Jing Ruan, Kam W Leong*, and Xianqun Fan**

© 2021 Wiley-VCH GmbH
Supporting Information

Title: Dose-dependent Carbon Dot-induced ROS Promote Uveal Melanoma Cell Tumorigenicity via Activation of mTOR Signaling and Glutamine Metabolism

*Author(s), and Corresponding Author(s)**

Yi Ding, Jie Yu, Xingyu Chen, Shaoyun Wang, Huixue Wang, Guangxia Shen, Renbing Jia, Shengfang Ge, Jing Ruan, Kam W Leong*, Xianqun Fan**

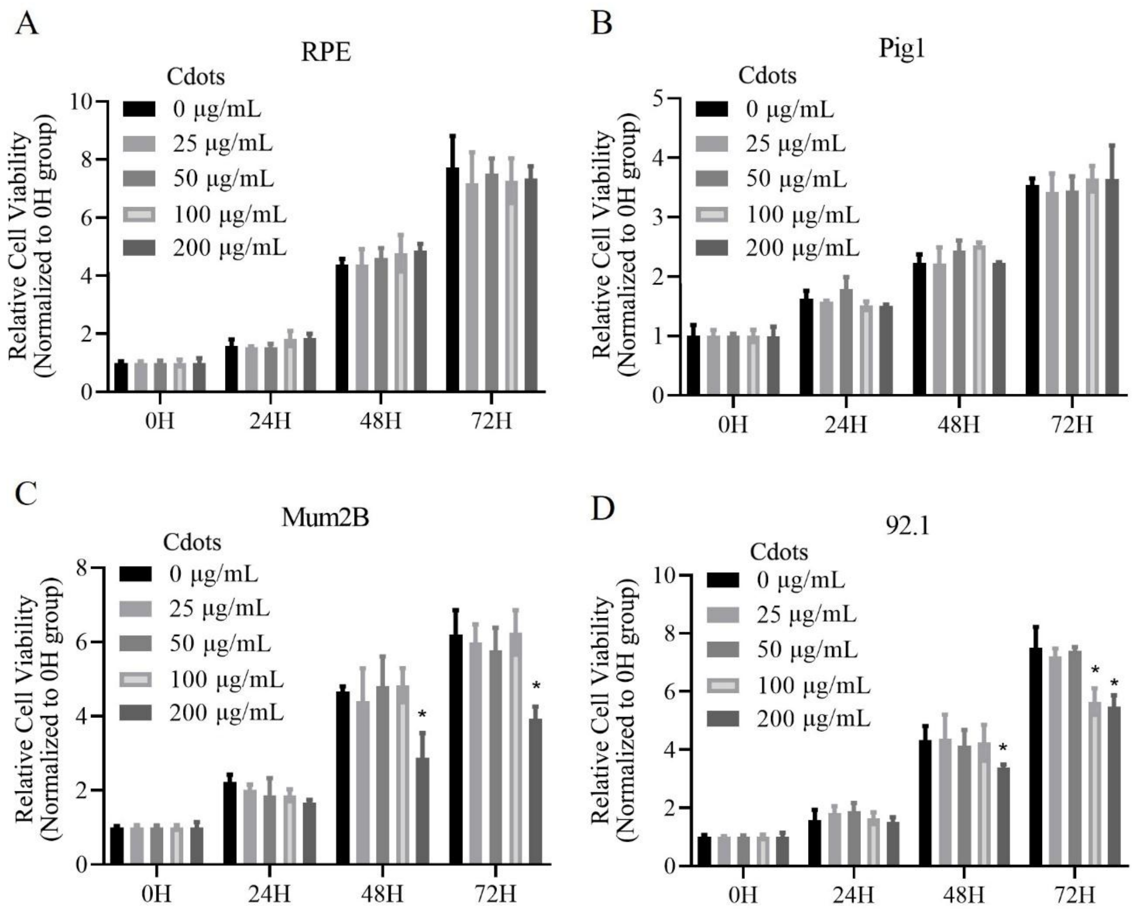

Figure S1. Effects of Cdots on normal and UM cell viability over 72 h measured by CCK8 assay. Exposure of (A) normal RPE cells and (B) PIG1 cells to Cdots at 0-200 μg/mL had no effect on cell proliferation. Exposure of (C) UM Mum2B cells to Cdots at 200 μg/mL and (D) UM 92.1 cells to Cdots at 100 and 200 μg/mL inhibited cell proliferation. *P < 0.05, **P < 0.01.

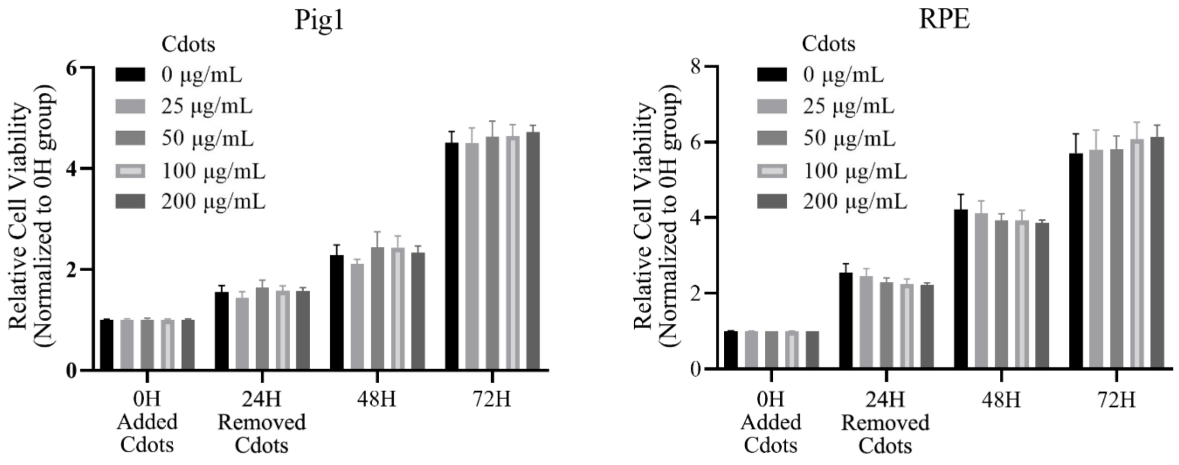

Figure S2. Effects of 24 h exposure of normal cell to Cdots on cell viability measured by CCK8 assay. Exposure of normal RPE cells and Pig1 cells to Cdots at 0-200 μg/mL during the second 24 h period had no effect on cell proliferation.

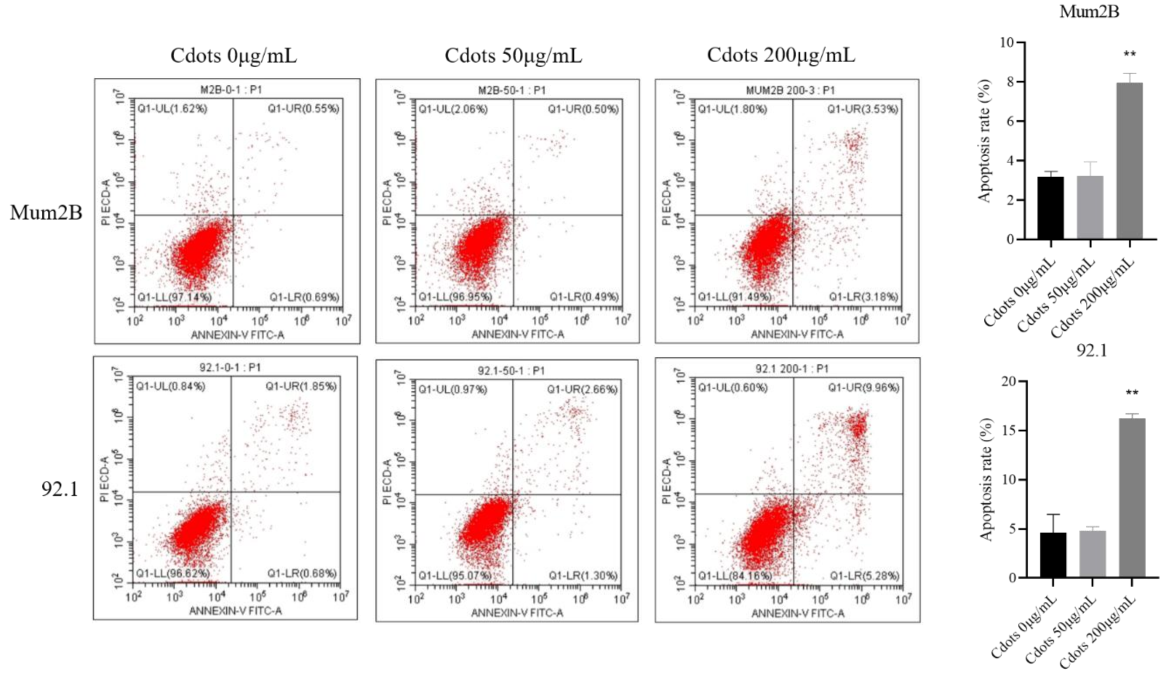

Figure S3. Effects of Cdots on UM cell apoptosis after 24 h exposure measured by FACS assay. Exposure of Mum2B cells and 92.1 cells to Cdots at 50 μg/mL had no effect on cell apoptosis. Exposure of Mum2B cells and 92.1 cells to Cdots at 200 μg/mL increased cell apoptosis. *$P < 0.05$, **$P < 0.01$.

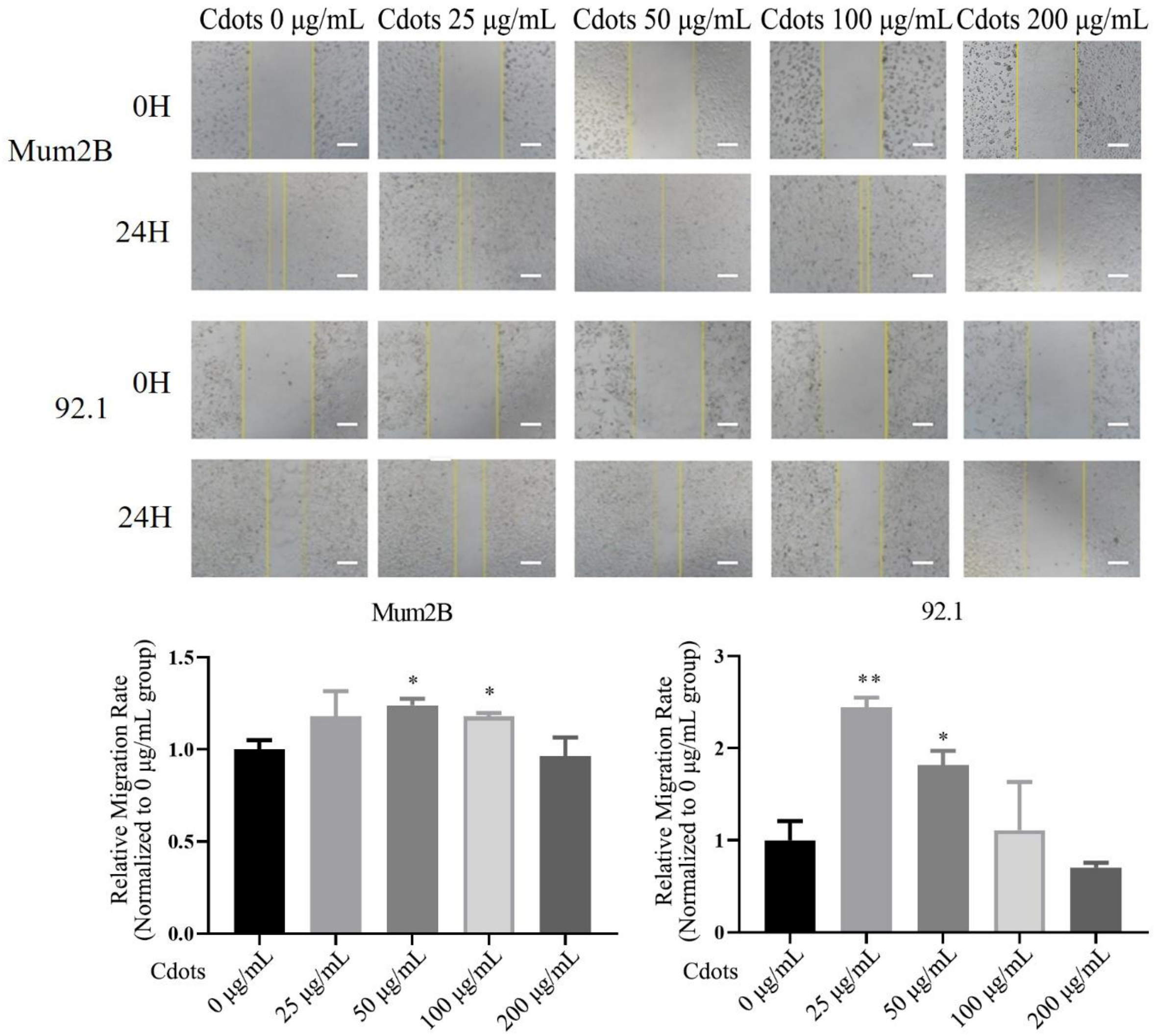

Figure S4. Cdots promote UM cell migration. Cell scratch assay results. Cdots at 50 and 100 μg/mL promoted Mum2B cell migration; Cdots at 25 and 50 μg/mL promoted 92.1 cell migration. *P < 0.05, **P < 0.01. Scale bars, 100 μm.

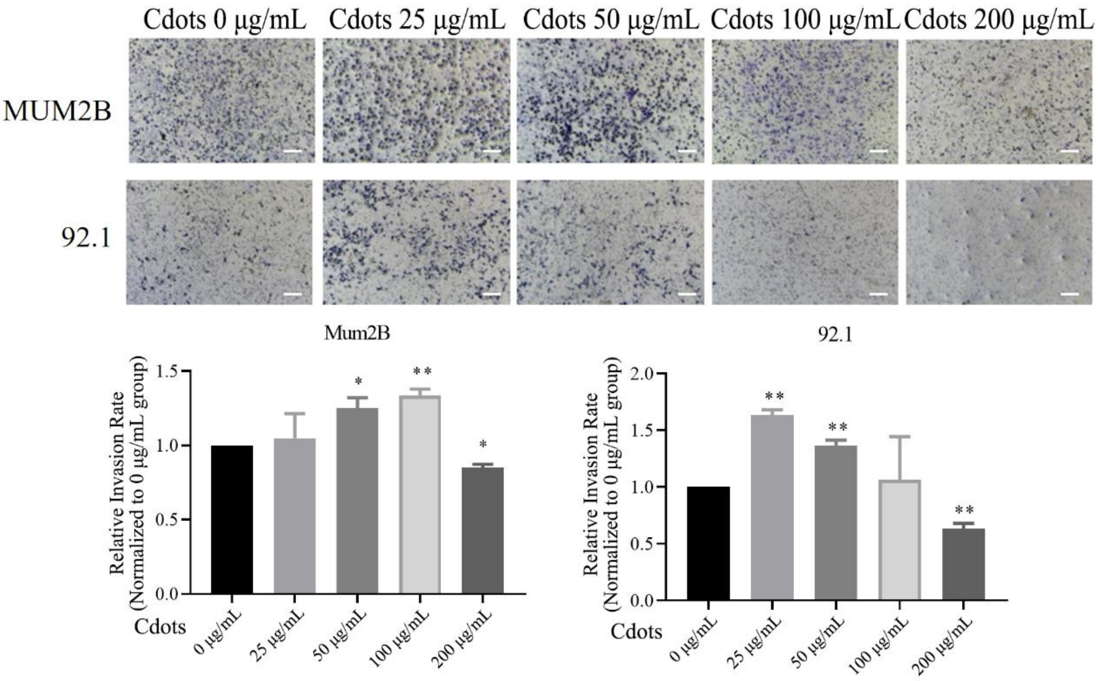

Figure S5. Cdots promote UM cell invasion. Transwell assay images. Cdots at 50 and 100 μg/mL promoted Mum2B cell invasion; Cdots at 25 and 50 μg/mL promoted 92.1 cell invasion. *P < 0.05, **P < 0.01. Scale bars, 100 μm.

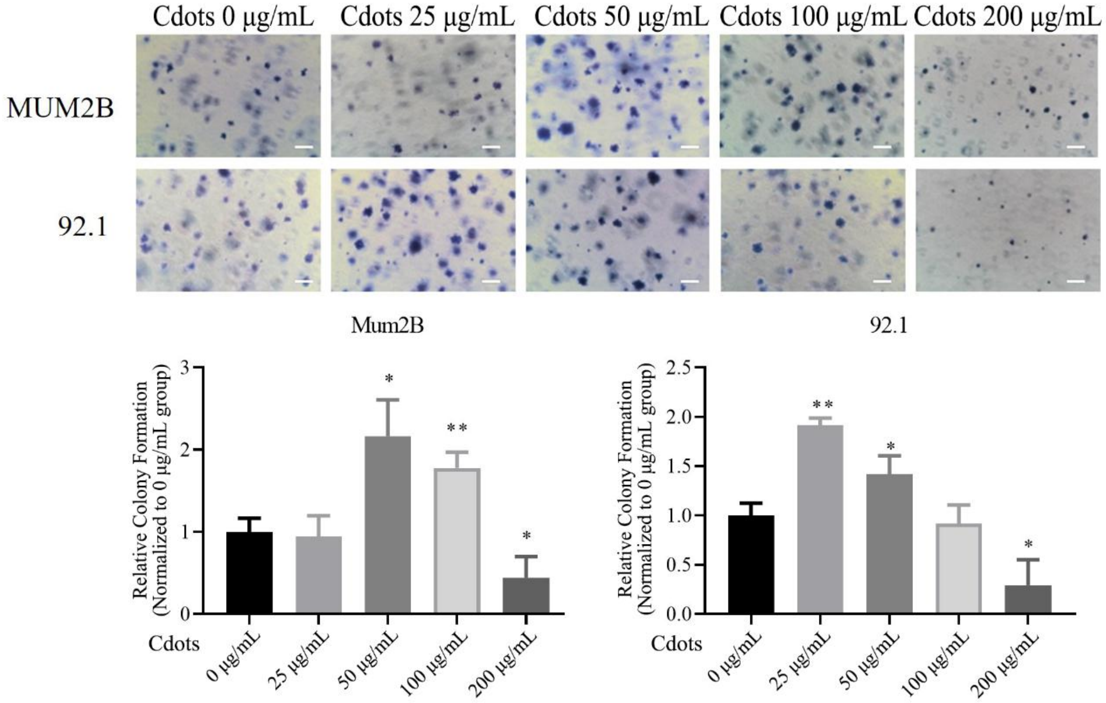

Figure S6. Cdots promote UM cell tumorigenesis *in vitro*. Soft Agar assay images. Cdots at 50 and 100 μg/mL promoted Mum2B colony formation; Cdots at 25 and 50 μg/mL promoted 92.1 colony formation. *P < 0.05, **P < 0.01. Scale bars, 200 μm.
